# Revolutionizing Glioblastoma Treatment: A Comprehensive Overview of Modern Therapeutic Approaches

**DOI:** 10.3390/ijms25115774

**Published:** 2024-05-26

**Authors:** Karol Sadowski, Adrianna Jażdżewska, Jan Kozłowski, Aleksandra Zacny, Tomasz Lorenc, Wioletta Olejarz

**Affiliations:** 1 The Department of Histology and Embryology, Medical University of Warsaw, Chalubinskiego 5, 02-004 Warsaw, Poland; karol.sadowski@wum.edu.pl (K.S.);; 2 Department of Biochemistry and Pharmacogenomics, Faculty of Pharmacy, Medical University of Warsaw, 02-091 Warsaw, Poland; wioletta.olejarz@wum.edu.pl; 3 Centre for Preclinical Research, Medical University of Warsaw, 02-091 Warsaw, Poland; 4 The Department of Anatomy and Neurobiology, Medical University of Gdansk, Dębinki 1, 80-211 Gdansk, Poland; adrianna.jazdzewska@gumed.edu.pl; 5 Department of Radiology I, The Maria Sklodowska-Curie National Research Institute of Oncology, Roentgena 5, 02-781 Warsaw, Poland

**Keywords:** glioblastoma, cancer, genetics, immunotherapy, CAR-T, immune checkpoint inhibitors

## Abstract

**Simple Summary:**

Glioblastoma is one of the most dangerous tumors of the central nervous system, and still cannot be fully overcome. We still mainly rely on standard treatments combining chemotherapy, radiotherapy, and surgery in various combinations. However, advances in technology, particularly biotechnology, are allowing the introduction of new, more precise therapies. These are more personalized, targeting a specific stage of tumor development or a particular cell population. The treatment of glioma is one of the areas in which we see more and more personalized medicine. In this article, we have tried to describe the current state of knowledge regarding the use of modern therapies in treating glioblastoma and the primary concerns.

**Abstract:**

Glioblastoma is the most common malignant primary brain tumor in the adult population, with an average survival of 12.1 to 14.6 months. The standard treatment, combining surgery, radiotherapy, and chemotherapy, is not as efficient as we would like. However, the current possibilities are no longer limited to the standard therapies due to rapid advancements in biotechnology. New methods enable a more precise approach by targeting individual cells and antigens to overcome cancer. For the treatment of glioblastoma, these are gamma knife therapy, proton beam therapy, tumor-treating fields, EGFR and VEGF inhibitors, multiple RTKs inhibitors, and PI3K pathway inhibitors. In addition, the increasing understanding of the role of the immune system in tumorigenesis and the ability to identify tumor-specific antigens helped to develop immunotherapies targeting GBM and immune cells, including CAR-T, CAR-NK cells, dendritic cells, and immune checkpoint inhibitors. Each of the described methods has its advantages and disadvantages and faces problems, such as the inefficient crossing of the blood–brain barrier, various neurological and systemic side effects, and the escape mechanism of the tumor. This work aims to present the current modern treatments of glioblastoma.

## 1. Introduction

Gliomas are one of the most frequent brain tumors, and account for 30% of all cases and around 80% of malignant brain tumors [1]. The global incidence of gliomas is approximately 3–4 cases per 100,000 individuals per year in the United States. IDH-wild-type glioblastoma (GBM) is the most common yet aggressive type of glioma and accounts for up to 45–50% of all adult-type diffuse gliomas. The risk of developing gliomas increases with age, and the majority of cases are diagnosed over the age of 50. GBM is seen more often in men than in women, with a gender ratio of approximately 1.5:1 [2]. Genetic risk factors include inherited disorders such as Li Fraumeni Syndrome, Turcot syndrome, Neurofibromatosis type 1, and several other specific genetic mutations predisposing to the development of GBM: *TP53, PTEN, EGFR, NF1* mutations, and chromosomal aberrations such as trisomy of chromosome 7 and monosomy of chromosome 10 [3,4,5]. The prognosis for patients with GBM remains poor, with an approximate median survival of 14 months [6].

Traditional therapeutic approaches for GBM include surgical resection, radiotherapy (RT), and chemotherapy, often with temozolomide (TMZ). These treatments, while standard, face significant challenges, particularly due to the tumor’s ability to infiltrate extensively into surrounding brain tissue and the presence of the blood–brain barrier (BBB), which limits the effectiveness of many systemic therapies. Furthermore, GBM’s heterogeneity and high recurrence rate pose additional hurdles in treatment. Adverse effects of RT are commonly categorized into three groups: acute, early-delayed, or late-delayed, based on the timing of symptom onset relative to treatment administration. Acute effects manifest shortly after treatment initiation, early-delayed effects emerge within a few weeks to months, while late-delayed effects may surface months to years later [7]. Developments in immunotherapy, molecular biology, and technology are allowing the search for new therapies for the treatment of GBM. Among these, targeted therapies and personalized medicine approaches have been gaining attention. These include drugs that target specific molecular alterations in GBM cells, such as the *EGFR*, *PDGFRA*, or *MGMT* gene mutations. Innovations in immunotherapy, such as checkpoint inhibitors, vaccines, and adoptive cell transfer techniques, are also being explored. Additionally, advanced technologies like tumor-treating fields (TTFs), a non-invasive treatment modality, are being evaluated for their efficacy and safety in treating GBM. The leading countries in a number of clinical trials in the field of GBM are the USA, China, Germany, Japan, Italy, France, and Canada [8]. The list of them with crucial information is attached as Supplementary Table S1. This article describes some targeted and modern approaches to GBM treatment, discussing their advantages and inconveniences.

### 1.1. Histopathology

Gliomas probably originate from glial progenitor cells that have relevant subtypes distinguished by malignancy grades and histological and genetic methods [9,10]. GBMs frequently manifest indications of increased pressure within the cranium due to their mass effect, often presenting as notably large upon initial diagnosis, and can occupy a significant portion of a cerebral lobe [11]. The majority of cerebral hemisphere GBMs are situated intraparenchymally, primarily within the white matter [12]. Pathology records generated from neurosurgeons’ observations during surgery consistently describe these tumors as having indistinct boundaries, with a variable-colored cut surface. The tumors exhibit grayish masses at their periphery and central areas displaying yellowish necrosis. Following formalin fixation, GBMs become fragmented and soft, displaying a gray to pink border adjacent to the brain tissue [13,14]. Certain tumors also feature macroscopic cysts containing cloudy fluid, representing liquefied necrotic tumor material [14,15]. GBM exhibits extensive morphological variability, reflecting its historical term “glioblastoma multiforme” [14]. At the core of the tumor, a spindle-shaped, atypical, and pleomorphic cell population is observed, while traces of low-grade neoplastic astrocytes are also often identifiable to varying degrees [16]. Cellular pleomorphism encompasses a range of types such as small, undifferentiated, giant, epithelioid, spindled, gemistocytic, lipidized, and sarcomatoid cells. Some tumors may prominently display specific patterns, contributing to the characterization of distinct subtypes within the spectrum of GBMs [14,15,17,18,19]. The main cellular feature of malignant glial cells is local tissue invasion, which typically occurs along deep white matter tracts [20]. Most GBMs exhibit nuclear atypia, greater cellularity, multiple mitotic figures, and a high degree of nuclear pleomorphism. Significant variation in cellularity is often seen in different parts of the tumor and can lead to misdiagnosis if the specimens are obtained by stereotactic needle biopsy [14,15,17,18,19,21].

### 1.2. Glioblastoma Classification

The World Health Organization (WHO) established the new classification of glioma tumors in 2021. According to this classification, there are three types of adult-type diffuse gliomas, namely astrocytoma (isocitrate dehydrogenase-*IDH*-mutant, grades 2–4), oligodendroglioma (*IDH-mutant*, *1p/19q-codeleted*, grades 2–3), and glioblastoma (*IDH-wild-type*, grade 4). Diagnosis of IDH-wild-type glioblastoma within the context of an IDH-wild-type diffuse and astrocytic glioma is dependent on the presence of several criteria—microvascular proliferation, necrosis, TERT promoter mutation, EGFR gene amplification, or +7/−10 chromosome copy number changes [22]. Wild-type IDH1/2, TERT promoter mutation, chromosome 7 gain, chromosome 10 loss, and epidermal growth factor receptor (EGFR) amplification are currently considered to be molecular hallmarks of glioblastoma, even despite the presence of histopathological features typically found in lower-grade diffuse gliomas [23].

**Figure 1:**
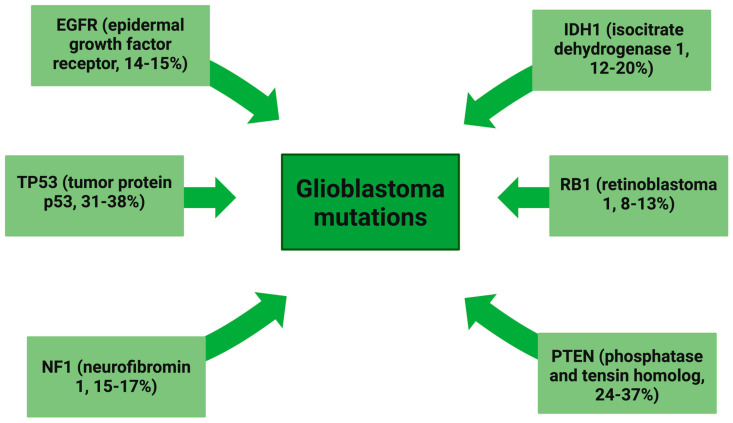
Glioblastoma mutations.

Contrast-enhanced magnetic resonance imaging (MRI) is the preferred diagnostic technique for GBM. Research has indicated that the tumor diameter typically falls within the range of 5 to 10 cm in most instances upon diagnosis [14]. The tumor usually involves corpus callosum and grows into occipital and temporal lobes bilaterally, resulting in a butterfly pattern on imaging; hence the name “butterfly glioma.” [24]. A surgically excised tumor obtained during the procedure is necessary for a conclusive diagnosis through histopathological examination [25]. In cases where tumor resection is not possible, or if metastatic GBM is suspected, fine-needle aspiration biopsy is performed on accessible sites [26]. It is typically advised to conduct examinations to determine the presence of glial fibrillary acidic protein (GFAP), *IDH* mutation status, and the methylation status of the O6-methylguanine-DNA methyltransferase *(MGMT)* promoter. GBM may be diagnosed as a secondary GBM, where the initial tumor is a low-grade glioma that progresses to high-grade glioma, and primary glioblastomas (appearing de novo), as shown in Figure 1 [27,28]. The overall median survival (mOS) rate of GBM patients is 15 months, and 5-year survival is less than 4% of all cases [29]. GBM is frequently diagnosed in the United States at above 60 years of age and rarely appears in pediatric cases [30]. The most common location of GBM is the supratentorial region (frontal, temporal, parietal, and occipital lobes), with the highest incidence of occurrence in the frontal lobe [31]. IDH mutations are frequent in lower-grade gliomas and secondary GBMs [22,32]. *IDH-wild-type* GBM appears de novo, without any precursors, and is more often diagnosed among older patients [33]. It is noteworthy that glioma patients with *IDH1 or IDH2 mutations* tend to have better prognoses than those with the wild-type form of these genes (median survival in GBM-*IDH wild-type*: 15 months [34]; *IDH mutant*: 31 months). Patients with high-grade gliomas carrying *IDH1* or *IDH2 mutations* are also usually younger than those with *IDH1/IDH2-wild-type* glioma [9]. It is important to keep in mind that the previous classification of central nervous system (CNS) tumors by the WHO in 2016 defined *IDH1/IDH2* high-grade gliomas as GBM. So, when analyzing results from studies published before 2021 or clinical trials initiated before this date, one should be aware that the group of patients previously classified as having GBM may also include those with *IDH1/IDH2 mutations*, which would not be diagnosed as GBM according to the current WHO classification [35].

The most common mutations in GBM tumors are those detected in *TP53* (tumor protein 53, frequency of mutation: 31–39%), *PTEN* (phosphatase and tensin homolog, mutated in 24–37% of tumors), *IDH1* (mutated in 12–20%), *EGFR* (14–15%), and *NF1* (neurofibromin 1, 15–17%). However, mutations in *PTEN* and *TP53* are not considered as GBM-specific markers, due to the common occurrence in other types of cancer. In the case of *EGFR*, the epidermal growth factor receptor variant III *(EGFRvIII)* mutant lacks 267 amino acids (exons 2 to 7 of the *EGFR gene*) in an extracellular domain resulting in in-frame deletion, which is characteristic of glioblastoma. Other frequently mutated genes are *PIK3CA* and *PIK3R1* [36].

Another approach based on Integrated Genomic Analysis distinguished primarily four relevant subtypes of GBM—classical, proneural, neural, and mesenchymal [37]. The neural subtype has been recently found to represent non-cancerous cells collected together with the tumor sample, and subsequently has been dismissed by Wang et al. Classical GBM is characterized by frequent *EGFR gene* amplification and mutations in the *TP53 gene* [37]. The mesenchymal GBM tumors were found to harbor *NF1, PTEN*, and *RB1* mutations, as seen in Figure 1. Histology has shown that the classic subtype had similar features to astrocytes, the proneural subtype had similar features to oligodendrocytes, and the mesenchymal subtype had similar features to astroglial and microglial morphologies [38].

## 2. Standard Methods of GBM Treatment

### 2.1. Current Guidelines

The current standards of treatments are based mostly on the new Stupp Protocol. The maximal surgical resection of the tumor followed by concurrent RT of 60 grays (Gy) in 30 fractions combined with TMZ (75 mg/m^2^/day for six weeks) chemotherapy and six maintenance cycles of TMZ (150–200 mg/m^2^/day for the first five days per month) is the standard therapy (new Stupp Protocol) for newly diagnosed GBM patients [39,40,41]. This approach gives the patient mOS rate of 14.6 months [42]. It was shown that surgery combined with chemotherapy and RT was more efficient than surgery with RT only (mOS 14.6 months vs. 12.1 months) [43]. 

Another approach involves the use of angiogenesis inhibitors (bevacizumab), which have been demonstrated to improve progression-free survival (PFS) (*p* = 0.01) and mOS (*p* = 0.04) in the bevacizumab-treated group compared to the non-bevacizumab treated group [44]. However, this treatment is used only in recurrent GBMs; the standard treatment remains the new Stupp protocol. Bevacizumab is a monoclonal antibody (MAb) that binds to circulating vascular endothelial growth factor A (VEGF-A) and prevents interaction with the VEGF receptors (VEGFRs), which precludes proliferation and vascular growth within the tumor [45]. Montemurro et al. showed that in the group of 5736 patients, only 2279 underwent a reoperation of tumor and showed improved mOS of 18.5 months [46]. Hypofractionated stereotactic radiotherapy (HFSRT) after stereotactic radiosurgery (SRS) was proven to increase the mOS to 11 months among patients with glioma [47]. Second-line chemotherapy is also possible, but the drug should be chosen individually; no standard treatment exists [48].

### 2.2. Tumor Heterogeneity—A Barrier to Effective GBM Treatment

**Figure 2:**
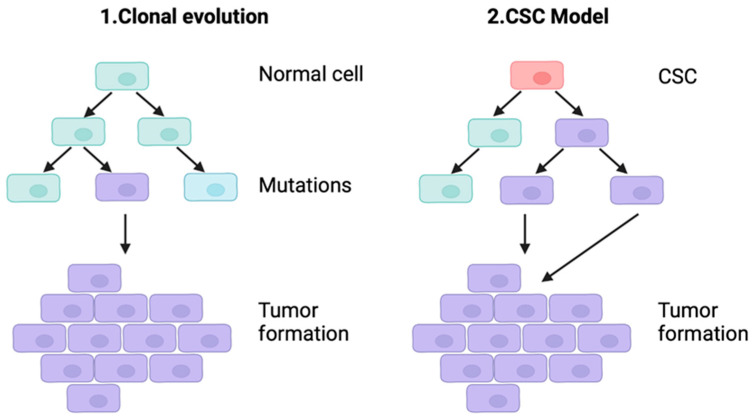
Two models of GBM tumors’ formation: clonal evolution (1) and cancer stem cells (CSCs) (2).

One of the greatest challenges in GBM treatment is its extensive heterogeneity, which refers to intertumor (population-level differences) and intratumor heterogeneity (individual tumor differences) [49]. Escape mechanisms that evade immune surveillance are particularly relevant due to the possible complications within standard and immunotherapeutic treatments. It is known that chemosensitivity in malignant gliomas differs among cellular subpopulations [50]. Hence, it implicates challenges for drug discovery and possible treatments as the population of cells may bottleneck, resulting in subsequent tumor progression [51]. Two major models can explain the cellular heterogeneity of glioblastoma: clonal evolution and the cancer stem cells (CSCs) hypothesis [52], as depicted in Figure 2. 

The clonal evolution model proposes that normal cells undergo a series of mutations, eventually giving rise to cancer cells that form tumors in a clonal manner [53]. The CSCs model suggests that embryonic or adult stem cells or progenitor cells undergo mutations that produce CSCs. The CSCs model suggests that mutations in embryonic or adult stem cells, or in early-stage cells, can give rise to CSCs, which possess the ability to differentiate into tumor cells [54].

Recognizing GBM tumor heterogeneity is crucial. This importance is highlighted by the potential for treatments, like TMZ, to induce mutations in the mutS homolog 6 (*MSH6*) gene that were not initially present in the GBM tumor [55]. There are many techniques to assess tumor heterogeneity. One is multiple tissue sampling, which found genome-wide intratumor variability in GBM [56], and the fluorescent in situ hybridization (FISH) method. Moreover, new generation sequencing and transcriptome analysis, including single cell sequencing, enable the assessment of the mutations in one cell, which gives an insight into tumor evolution and heterogeneity [57]. Another method of the microfluidic platform, microfluidic image cytometry, is capable of single-cell level proteomic analysis, enabling the prediction of tumor progression and survival [58].

## 3. Innovative Approaches to GBM Treatment

### 3.1. Gamma Knife

Gamma knife radiosurgery (GKRS) is a therapy developed in the late 1960s by Prof. Lars Leksell [59]. It delivers a high radiation dose to the tumor in a single session. Among various stereotactic radiosurgery techniques, GKRS is one of the most popular worldwide. The device is constructed from an array of about 200 sources of cobalt-60. The decay of the isotope in many arrays enables the creation of photon beams (gamma rays), which are aligned and converged at a single point, generally the tumor lesion [60]. Thanks to this method, high levels of radiation may be delivered to a specific target, without excessive irradiation of normal brain tissue, which often occurs in the optic way. One advantage of GKRS is that it does not generally cause serious adverse effects among patients. Moreover, it is considered to be an increasingly popular salvage treatment modality with minimal burden for recurrent gliomas [61]. The disadvantages of this therapy include difficulty in targeting tumors that are bigger than 40 mm or cannot be well visualized. GKRS is also not recommended in newly diagnosed GBM, as it shows a lack of sensitivity [62]. Therefore, gamma knife radiosurgery is primarily used as an adjuvant treatment to chemotherapy in patients after tumor recurrence. Kondziolka et al. demonstrated the survival benefit of GKRS in 1996 [63]. The study included 64 GBM patients and resulted in 26 months mOS after initial diagnosis. Better outcomes were observed when radiosurgery was performed at the time of tumor progression [63]. However, Souhami et al., in a randomized, two-arm trial with 203 patients with supratentorial GBM (minimum tumor size was 40 mm), observed no difference in median survival between patients with GBM treated with radiation only and with radiation combined with GKRS [64]. One possible explanation of this is the moment of GKRS administration, since Souhami et al. administered it one week before initiating radiation therapy. Therefore, GKRS is mainly used as an adjuvant therapy and not as an initial management [65].

Kong et al. treated 65 recurrent GBM patients with both GKRS and SRS compared to a historical control group and obtained an outcome of 23 months mOS after diagnosis [66]. Skeie et al. conducted a retrospective analysis of 77 recurrent GBM patients, among whom 51 underwent GKRS. It proved GKRS is an alternative treatment to open surgery at the time of recurrence of smaller tumors. The time of tumor progression after the second intervention was significantly longer for patients receiving GKRS compared to those with reoperation only (12 and 6 months, respectively) [67]. Gamma knife radiosurgery is a well-tolerated adjuvant therapy that provides a high local tumor control rate, but only to a subset of GBM patients with recurrent and small-sized tumors [62]. The interesting use of GKRS is termed the leading-edge radiosurgery (LERS) method [68]. This method is based on the observation that GBM cells migrate through the brain along well-defined white matter pathways. By targeting these pathways, LERS aims to disrupt and destroy them, thereby limiting the spread of glioma cells and improving patient outcomes. This method has demonstrated significant potential in clinical studies; it has been shown to be a safe and effective adjunctive therapy for patients with newly diagnosed GBM. The successful application of LERS in early treatment stages reinforces its role as a critical and safe component of GBM management [68].

### 3.2. Proton Beam Therapy

Proton beam therapy (PBT) delivers a high radiation dose without destroying critical organs and tissues surrounding the target area. The difference between proton and conventional photon RT is that protons lose energy at the end of their path, while photons deposit it all the way. Thus, the administration of PBT enhances precision of the radiation procedure and considerably reduces the area of healthy tissue irradiation. The potential of PBT in fighting intracranial tumors was demonstrated in meningioma patients, whose PFS and mOS significantly improved with PBT application [68,69]. These encouraging results led to clinical trials employing PBT in GBM patients. Mizumoto et al. described the outcome of a study in which 46 GBM patients were treated with PBT as a radiation method along with one of two adjuvant drugs: TMZ or nimustine hydrochloride. The combination of PBT and chemotherapy resulted in a median survival of 21.0 months for the nimustine group and a median survival of 25.7 months for the TMZ group [70].

In terms of dosage, PBT is tailored to the individual patient, considering factors such as the type and location of the tumor, its size, and proximity to critical structures. The goal is to deliver the maximum effective dose to the tumor while preserving healthy tissue functionality, a principle known as the therapeutic ratio.

PBT stands out for its precision and reduced side effects, but faces limitations including high costs due to its complex technology involving cyclotrons or synchrotrons for proton acceleration and advanced targeting systems. This complexity leads to higher expenses than conventional RT. Moreover, its precision requires detailed planning and imaging, and there is ongoing research into its effectiveness compared to traditional therapies, highlighting a need for further trials.

In conclusion, PBT represents a significant advancement in RT, offering heightened precision and reduced side effects for cancer treatment. Its application in GBM and other challenging cancer types continues to be explored, with ongoing research aimed at maximizing its therapeutic potential while addressing its limitations.

### 3.3. Tumor-Treating Fields

Tumor-treating fields (TTFs) deliver alternating electric fields that disrupt cell division, using a specially designed device. These electric fields are of low intensity (1–3 V/cm) and intermediate frequency (100–300 kHz) [71,72]. TTFs are a part of current FDA and National Comprehensive Cancer Network guidelines both in newly diagnosed and recurrent GBM [71,73]. There was a debate whether TTFs should become a part of the standard of care. This proposition was rejected due to expensive costs and inconvenience for patients [41]. The device consists of special electrodes applied to patients’ scalps that creates a TTF [71]. The mechanism of the TTF’s outcome has not been thoroughly examined yet, but it is confirmed that alternating electric fields affect cells undergoing mitosis [74]. One assumption is that it prevents microtubules from forming a proper mitotic spindle [71]. Affected tumor cells have mitotic disruption, blocking cell proliferation and causing their death. TTFs barely affect nondividing cells [74].

Kirson et al. performed a clinical study in which the NovoTTF-100A system caused the mOS to be doubled compared to a median historical control value at that time (62.2 weeks vs. 29.3 ± six weeks) [75]. Based on these results, a phase III trial was conducted in 2012 in recurrent GBM patients [76]. TTFs were tried as a monotherapy and compared to chemotherapy. They gave similar mOS outcomes (6.6 to 6 months, respectively) [76,77]. An EF-14 trial with 695 randomized patients with newly diagnosed GBM who completed RT was also performed. It compared adjuvant TMZ-only and adjuvant TMZ with TTF patient groups. The results indicate that patients in the TMZ/TTF group experienced an extension of 2.7 months in PFS and an extension of 5 months in mOS [78].

### 3.4. Brachyterapy

Brachytherapy (BT) for GBM utilizes radioactive isotopes to administer ionizing radiation directly to the tumor site [7,79,80]. The radiation source is positioned either within the tissue or the region adjacent to the target, emitting radiation gradually, and is effective only over a limited distance. The meta-analysis performed by Barbarite et al. showed survival advantages of BT relative to the standard of care. BT could be a beneficial therapy for selected GBM patients combined with other therapies [79]. Similarly, Xiang et al. in their systematic review showed that that brachytherapy has acceptable safety and good post-treatment clinical efficacy for selected patients with recurrent GBM [80].

### 3.5. EGFR and VEGF Inhibitors

Epidermal growth factor receptor (EGFR) is a transmembrane tyrosine kinase that belongs to the erythroblastic leukemia viral oncogene homologue (ErbB) family of the receptor tyrosine kinases (RTKs). [81] EGFR activation in cancer cells promotes proliferation and protects transformed cells against apoptosis [82]. EGFR amplifications and mutations are detected in 40–60% of GBM cases. The most common mutational variant, EGFRvIII, occurs in about 50% of patients with EGFR amplification and leads to constitutively active EGFR [82].

#### 3.5.1. EGFR Inhibitors

EGFR is a member of the receptor tyrosine kinase family involved in the regulation of cell growth and division. The *EGFR* gene is located on chromosome 7p12 and encodes for the EGFR protein [83]. This receptor is activated by binding a variety of ligands, such as epidermal growth factor (EGF) and transforming growth factor-α (TGF-α) [84]. Upon ligand binding, EGFR undergoes dimerization and autophosphorylation to subsequently trigger a series of pathways like mitogen activated protein kinase (MAPK), AKT and Janus kinase (JNK) pathways [83]. That leads to proliferation and differentiation of the cells, as well as the inhibition of apoptosis. The activation of the RAS/MAPK pathway, for example, results in the signaling cascade and stimulation of extracellular signal-regulated kinase ½ (ERK½) [84]. These proteins, in turn, phosphorylate a lot of downstream substrates and are transported to the nucleus. Consequently, the activity of specific transcription factors may be regulated. EGFR is often overexpressed and/or mutated in various cancers. EGFR inhibitors target this receptor and block its activation.

Gefitinib and erlotinib are inhibitors of EGFR tyrosine kinase activity. However, not only gefitinib, but also combinations of erlotinib with other therapies (such as TMZ or RT), do not result in improved mOS [85,86,87]. The outcomes do not appear to correlate with gene amplification and expression of wild-type or mutant EGFR. Toxicities such as thrombocytopenia, anemia, lymphopenia, and febrile neutropenia have also been observed in newly detected GBM during treatment with erlotinib [86]. The main challenges in this therapy are low penetration across the BBB and the escape mechanisms (e.g., the phosphoinositide-3-kinase (PI3K) pathway) [88].

The failure of erlotinib and gefitinib in GBM treatment induced the development of second-generation EGFR tyrosine kinase inhibitors (TKIs) such as afatinib and dacomitinib, which bind irreversibly to more than one receptor of the ErbB family receptors [89]. Even though they are considered to excellently inhibit both mutant and wild-type EGFR, they provided no special benefits in clinical trials. Interestingly, a phase II trial conducted by Sepúlveda-Sánchez et al., which examined the use of dacomitinib, showed a difference in mOS between patients with EGFRvIII mutation and without it (6.7 to 7.8 months, respectively) [90,91]. One of the reasons may be low BBB penetration [92]. Moreover, lapatinib, a dual human epidermal growth factor receptor 2 (HER2) and EGFR kinase inhibitor, failed to exhibit therapeutic gain in recurrent GBM in a group of 16 patients, among whom 4 had EGFRvIII expression and 6 had PTEN loss [93]. Consequently, third-generation EGFR TKIs (e.g., AZD3759 or osimertinib) have been developed. They showed excellent BBB penetration. However, clinical trials with osimertinib or AZD3759 in GBM patients have not been completed yet [88].

Another type of EGFR inhibition is antibody-based therapy. MAbs, such as cetuximab and nimotuzumab, stand out for their high specificity and affinity [94,95]. Their single use so far did not show improvement over existing therapies [96]. A phase II trial combining a triple therapy regimen with cetuximab, bevacizumab, and irinotecan has failed [97]. One of the newest of the developed MAbs is depatuxizumab mafadofin (depatux-m). It was created mainly against EGFRvIII in mice, but binds also to EGFR wild-type, which provided the high occurrence. Lassman et al. conducted a phase III trial with 639 patients with EGFR-amplified newly diagnosed GBM. The two groups included RT, TMZ and depatux-m or RT, TMZ, and placebo. The study failed to achieve an improved mOS, but PFS was longer for depatux-m than placebo (8.3 compared to 6 months) [98].

#### 3.5.2. VEGF Inhibitors

Vascular endothelial growth factor (VEGF) is a signaling protein that stimulates the growth of new blood vessels in the angiogenesis. It operates through receptors on the surface of cells, primarily endothelial cells, which line the inside of blood vessels. These receptors belong to the RTK family, identified as VEGFR1, VEGFR2, and VEGFR3. Upon binding VEGF, these receptors dimerize and autophosphorylate, initiating a cascade that promotes endothelial cell proliferation and migration. VEGF inhibitors block the interaction between VEGF and these receptors.

**Figure 3:**
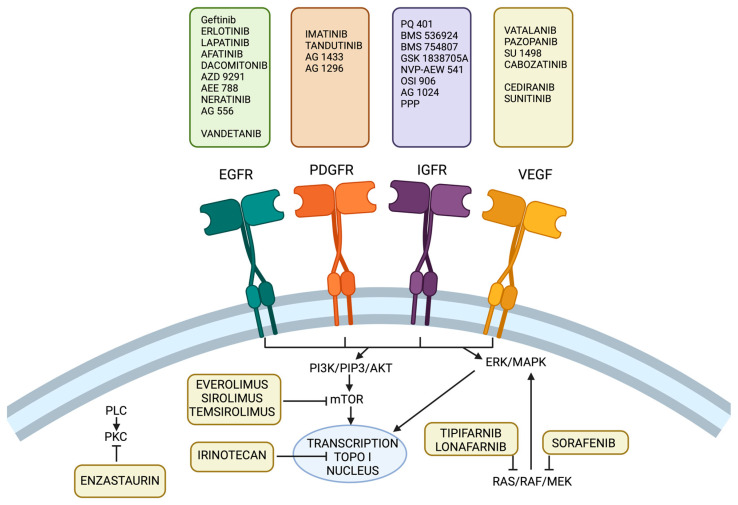
The current therapies inhibit EGFR, PDGFR, IGFR, and VEGF receptors. Epidermal growth factor receptor (EGFR), platelet-derived growth factor receptor (PDGFR), insulin-like growth factor (IGFR), vascular endothelial growth factor (VEGF).

Bevacizumab has been approved as a second-line agent for the treatment of GBM [99]. In contrast to the European Medicines Agency (EMA), the Food and Drug Administration (FDA) accepted bevacizumab for use in recurrent GBM in 2009 [100,101]. Bevacizumab improves PFS. In a phase II study of bevacizumab and irinotecan, the 6-month PFS was 46% and the 6-month mOS was 77% [102]. Chinot et al. showed that respective mOS rates at one year with bevacizumab and placebo were 72.4% and 66.3%, respectively [103]. The mechanism and summary of the EGFR, platelet-derived growth factor receptor (PDGFR), insulin growth factor receptor (IGFR), and VEFG are provided in Figure 3. [104,105].

### 3.6. Multiple RTK Inhibitors

RTKs play crucial role in cell cycle regulation as well as its proliferation and differentiation. The dimerization of their extracellular regions results in activating the intracellular tyrosine kinase domain. This leads to the autophosphorylation of the cytoplasmic tail and induces downstream signaling pathways, which impair apoptosis and increase angiogenesis [61]. Since RTKs are amplified and coactivated in up to 50% of GBM cases, they are considered as a potential therapeutic target [106]. Some studies suggested inhibiting multiple RTKs; their shared downstream signaling might improve the outcomes [104]. Vandetanib is one of the multitargeted RTK inhibitors that blocks EGFR and VEGFR [104]. Sunitinib, on the other hand, targets VEGFR and PDGFR. Imatinib is the most common small-molecule multiple RTK inhibitor. The 6-month PFS rate of Imatinib in a phase II trial was 16% [107]. In phase III clinical trial, it was examined in combination with hydroxyurea rather than as a monotherapy. The results of this trial highlighted its modest activity; the 6-month PFS was very similar (5% in the combination arm vs. 7% in the monotherapy arm) [108].

### 3.7. PI3K Pathway Inhibitors

The phosphoinositide-3-kinase/protein kinase B/mammalian target of rapamycin (PI3K/AKT/mTOR) pathway is considered one of the most dysregulated downstream pathways in GBM [109]. It is initiated by the RTKs (e.g., EGFR) [110]. PI3Ks are a family of intracellular lipid kinases, which induce activation of proteins targeting mTOR, a critical effector of cell signaling pathways [111]. This family is crucial for mediating such processes as cell proliferation and migration by ribosome biosynthesis and cytoskeletal organization [110,112]. The mutations or amplifications of the PIK3CA gene (the gene coding some subunits of PI3K) have been observed in 15% of GBM cases [110].

In addition, the loss of PTEN, which is an antagonist to PI3K, has been detected in about 40% of patients [110]. The loss of PTEN function might happen through several mechanisms including genetic mutation (production of dysfunctional PTEN protein or absence of it) such as nonsense, insertions, deletions, and frameshift. Chromosomal instability, and especially loss of heterozygosity (LOH), occur at the chromosome 10q23 locus, where PTEN is located. Other mechanisms, such as epigenetic silencing (hypermethylation for instance) and post-transcriptional regulations, play crucial roles in the loss of PTEN function. MicroRNAs (miRNAs) also play a role in the downregulation of PTEN by binding to the 3′ untranslated region (3′UTR) of its mRNA, inhibiting the translation [113]. Since the RTKs initiate the PI3K pathway [110], it was suggested that PI3K/AKT/mTOR pathway inhibition could be a considerable alternative for low-efficacy RTK inhibition [81]. Moreover, this pathway has been regarded as partially responsible for GBM invasiveness, since the activated AKT is able to phosphorylate mTOR. This, in turn, enables binding of cyclin D1 to cyclin dependent kinase (CDK) and stimulates cell division [114].

Buparlisib is PI3K inhibitor with significant penetration across the BBB. It showed inhibition of the growth of established human tumor xenografts in vivo [115]. A phase II trial with buparlisib revealed that 8% of patients achieved a PFS of 6 months [116]. Similar positive outcomes of the phase II trial testing Sonolisib efficacy also showed a median 6-month PFS of 17% [117]. Currently, ipatasertib, a pan-AKT inhibitor, is being tested in a phase I/II clinical trial (NCT02430363). Another approach to inhibiting this pathway is mTOR inhibitors, such as rapamycin (sirolimus) [111]. Several rapamycin analogs have been generated, e.g., temsirolimus and everolimus. They have better bioavailability, and all these molecules are FDA-approved agents [111]. They showed little antitumor activity in several phase II clinical trials. In one study, everolimus with TMZ and RT achieved 8.2 months of PFS compared to 10.2 months in the control [118].

### 3.8. Immunotherapy

#### 3.8.1. CAR-T Cells

**Figure 4:**
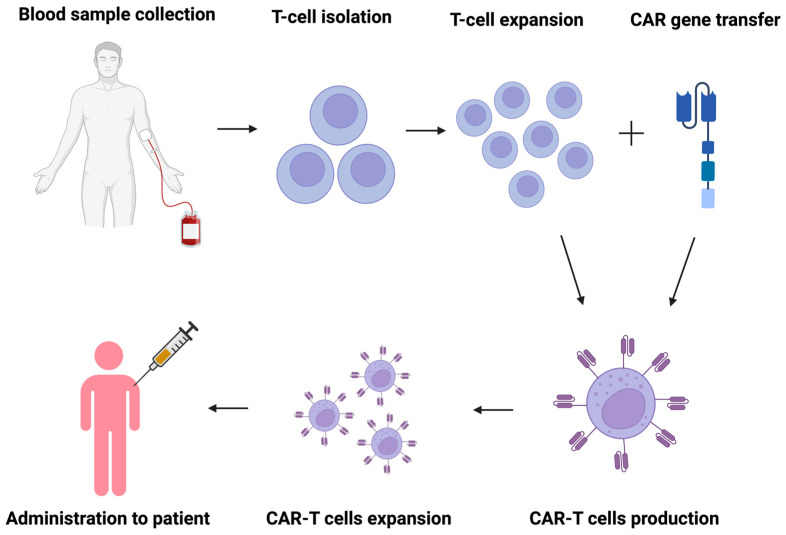
CAR-T cell production and administration. Chimeric antigen receptor T cells (CAR-T cells).

Genetically engineered T and NK cells expressing a chimeric antigen receptor (CAR) are cytotoxic cells for treating hematological malignancies and solid tumors [119,120,121,122,123]. They contain engineered synthetic receptors that mainly target lymphocytes against cells expressing a specific target antigen [124]. CAR cells combine the antigen-binding site of a MAbs with the costimulatory domains of a T cell. Their process of production and administration to the patient is shown in Figure 4. [121]. 

The crucial feature of CAR-T cells is their binding to target antigens on the cell surface independent of the major histocompatibility complex MHC receptor [119]. Their efficiency is limited by several factors, such as the immunosuppressive tumor microenvironment (TME), inadequate trafficking and infiltration of CAR-T cells, and their weak persistence and activity [125]. In recent years, the results in hematological malignancies have raised the curiosity of researchers who have taken a great interest in this therapy in solid tumors, including GBM [119,120,126].

Created CAR-T cells are administered to patients to destroy GBM cells expressing identified tumor antigen (TA). So far, clinical trials have been completed for several targets like EFGRvIII, tumor-associated antigen IL-13 receptor alpha 2 (IL13Ralpha2), and HER2. Another approach is to use EGFRvIII-specific CAR-T cells designed to produce bi-specific T cell engagers (BiTEs) [127]. This bi-specific MAb can connect T cells to wild-type EGFR cells, overcoming the resistance of heterogeneous GBM cells’ EGFRvIII to specific CAR-T cells’ EGFRvIII. They can successfully eliminate cancer cells and prolong the survival of mice orthotopically grafted with either GBM cell lines or patient-derived glioma neurospheres [127].

In contrast, administration of these CAR-T cells did not show survival benefits for CAR-T cell therapy [128]. Another aim for CARs is a receptor for IL-13 overexpressed in 58% of GBMs and is associated with poor prognosis [129]. It was shown that multiple infusions of CAR-T cells targeting IL13Ralpha2 in a patient with recurrent multifocal GBM regress the tumor and have no toxic effects observed in the resected tumor cavity followed by perfusion in the ventricular system. The patient presented with a significant clinical and radiographic response, although recurrence occurred 7.5 months after initiation of treatment [130]. Il13Ralfa2-specific CAR-T cells were effective and safe, with good clinical responses reported in the first-in-human pilot study [131]. Another target of clinical trials is HER2. This is an epidermal growth factor receptor expressed in normal epidermal cells, but it is overexpressed on several cancer cells, including GBM [132]. The successful administration of 1 × 10^8^ HER2-specific CAR-T cells, constructed with a CD28.ζ endodomain, was performed in GBM patients with no dose-limiting toxic effects. HER-2-targeted CAR-T cell therapy established an acceptable safety profile, with 1 of 16 patients achieving a partial response and seven demonstrating stable disease for 8 weeks to 29 months [133]. In addition to these three targets, there are several ongoing clinical trials, which include aims like B7-H3 (B7 family of immune checkpoint inhibitors), CD147 (cluster of differentiation 147, basigin), GD2 (disialoganglioside, a tumor-associated antigen), MMP2 (matrix metallopeptidase 2), and NKG2D (from the CD94/NKG2 family) ligands [125]. The preclinical studies focus on molecules like CAIX, CD70, CSPG4, EphA2, and TROP2 [125].

The main limitations to the use of CAR-T cell therapies to treat GBM are immunosuppressive TME, limited access across the BBB, toxicity, cytokine release syndrome, tumor lysis syndrome, or selective antigen loss. The heterogeneous expression in GBM often leads to the generation of escape variants resistant to CAR-T cell therapy. To minimize the risk of resistance to treatment, CAR-T lymphocytes may target multiple antigens or be combined with synergistic therapy. It was shown that under hypoxic conditions, CAR-T cells are more efficient in TME, which can be obtained by enhancing CAR-T cells in the hypoxia transcription factor HIF-1α subdomain [134].

#### 3.8.2. CAR-NK Cells

In CARs, more attention is paid to CAR-NK cells as a potential tool for cancer immunotherapy [135,136]. The advantage of this approach is the possibility of administration to an HLA-mismatched patient, allowing for an off-the-shelf therapy [135]. Another advantage is the lower risk of graft versus host disease, regulating adaptive immune responses through dendritic cells editing or not inducing cytokine release syndrome. So far, only one line has been approved by the FDA, i.e., NK-92 cells. Thus, most tests have been focused on this (e.g., NCT03383978). The current strategies to improve the antitumor activity of CAR-NK cells include the additional co-expression of the chemokine receptor CXCR4 to promote homing to the tumor site [137] and dual EGFR- and EGFRvIII-targeting CAR NK cells [138]. Both strategies demonstrated better tumor control in NSG mice wearing orthotopic GBM xenografts. The CAR2BRAIN clinical trial for recurrent glioblastoma is currently assessing the efficiency of intracranial injection of HER-2-specific NK-92 cells. After three dose levels, toxicities were not noticed (NCT03383978) [139]. Based on murine models, it was indicated that intravenously administered NK-92 cell line-derived CAR-NK cells could not cross the BBB without ultrasound disruption [140]. Thus, the preferential way to inject cells is the intracranial route of administration. The actual results of applying CAR-NK cells for patients with GBM result in favorable outcomes, but the results of clinical trials are needed to assess the efficacy of CAR-NK cell treatment of GBM [135,141].

#### 3.8.3. Dendritic Cell Vaccines

In recent years, the potential for combating and stabilizing oncological conditions through immunotherapy has been extensively discussed, with the proposal of vaccine therapies being particularly noteworthy. Numerous vaccines with diverse immunological foundations have been developed and tested for GBM treatment. Four commonly employed approaches for GBM vaccines include peptide and DNA vaccines, which utilize genetic information from the tumor itself and are highly specific; cellular vaccines, which are based on dendritic cells (DCs) prepared with tumor antigens; and mRNA-based vaccines that use viral vectors. Overall, the rationale behind these strategies is to elicit an immune response, addressing the tumor’s ability to evade the individual’s immune system [142,143]. DCs are antigen-presenting cells crucial to the active T cell response [144,145]. Typically, they are not found in the normal brain parenchyma but only in vascular components (e.g., choroid plexus or meninges). These spaces suggest the potency of migration pathways of peripheral DCs into the CNS [146,147,148].

**Figure 5:**
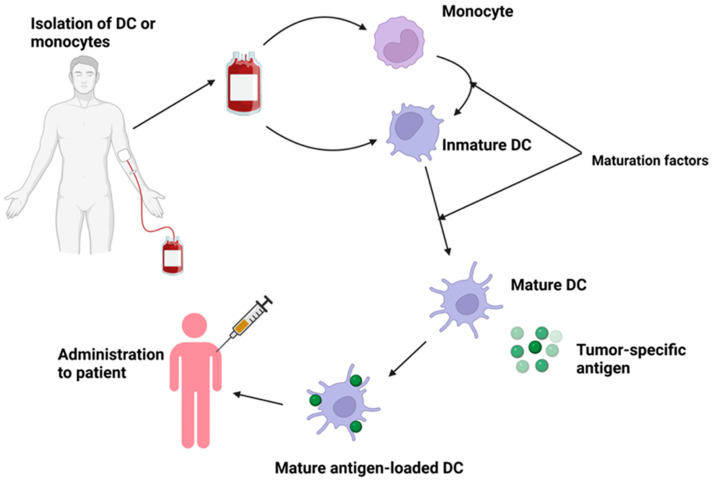
DC production and administration. Dendritic cells (DCs).

The therapy involves the isolation of DCs from the peripheral blood or induction of monocyte-derived DCs (MoDCs) ex vivo from peripheral blood. Figure 5 illustrates the process of obtaining and delivering DCs [149,150]. 

DC sources vary, but the primary source is usually MoDCs [151]. These cells are pulsed ex vivo with multiple tumor antigens to allow for the uptake, processing, and presentation of tumor antigens. The patients receive tumor-associated antigen (TAA)-loaded DCs, which can migrate to lymph nodes and present TAA-derived peptides on HLA molecules. Dendritic cell vaccines (DCVs) aim to increase antitumor T cell tumor-infiltrating lymphocytes (TILs) in the brain. Heimberger et al. vaccinated the mice with DCs pulsed with lysates from the 560 glioma cell line. They had been transfected with the murine homolog of the mutated EGFRvIII [152]. It was shown that the DCV results in antitumoral memory in surviving animals. Numerous animal studies have been performed in prophylactic [153,154,155] and curative DCV settings [156,157,158,159,160,161,162].

Most patients underwent cytoreductive surgery before the DCV due to the experimental aspects of this treatment. There is no clear consensus on whether the DCV should be administered before or after maximal resection, as a co-therapy with TMZ, after RT, or any other variants. It was shown that extent of resection was also a predictive aspect of survival [163,164]. Vaccination therapy has been indicated as being beneficial for the residual disease [164,165,166,167]. In contrast, Buchroithner et al. found that the extent of resection was not associated with survival [168]. There is a trend for better immunological responses in newly diagnosed patients due to less heavy pretreatment of those patients. The target for the DCV is the induction of antigenic target-directed immune responses.

For the future of modern treatment of GBM, the most informative are these DCV trials in phase III, which are assessing the clinical efficiency. Liu et al. showed long-term survivors and outcomes in all patients for 23.1 months (DCV group and control group). However, other data are not available yet. Based on phase II trials, it is possible to see some trends. Two randomized trials with 34 [169] and 25 [170] newly diagnosed patients showed mOS at 31.9 and 17 months. The clinical outcomes in the vaccination group were significantly improved compared to those in the control group (15 and 10.5 months). Similar results were achieved by another randomized phase II trial involving 41 newly diagnosed and recurrent patients with GBM [171]. They showed that DCV prolonged mOS to 13.7 months, compared to 10.7 months in the control group. The larger cohorts of patients in two randomized phase II trials with 76 [168] and 124 [172] newly diagnosed GBM patients did not show significant differences between the DCV and control groups (respectively, 18.8 vs. 18.9 months and 17 vs. 15 months). The positive clinical outcomes concluded from these two trials showed improved PFS for the vaccinated patients (11.2 vs. 9 months). Research on DCV often checks how other immune stimulants make it more effective. Batch et al. compared data on DCV with CMVpp65 mRNA transferred DC of newly diagnosed GBM patients, either admixed with GM-CSF or tetanus-diphtheria toxoid [173]. They reported an mOS of 41.1 (GM-CSF) patients and 41.4 months (compared to 18.5 for control patients receiving unpulsed DCs). There are still some questions without clear answers, e.g., it is unknown whether whole-tumor cell sources of TAA or molecularly defined TAA are better for inducing an antitumoral response. Actual results suggest the advantages of whole-tumor cell sources of TAA, which would suit extremely heterogeneous GBM [56]. Therefore, one drawback of using the whole tumor lysis approach in GBM is that other antigens may dilute particularly immunogenic antigens in the lysate. This may lead to less efficient and less effective uptake and presentation of immunogenic antigens by DCs to initiate a tumor response.

Another challenge in DCV therapy is efficient administration. The CNS strictly regulates the immune surveillance process by circulating cells of the immune system [174]. One of the possibilities is the intratumoral application of DCs. It was demonstrated that the efficacy of intratumoral application of GL261 lysate-loaded DCs is lower than that of subcutaneous application, and that the combination of both procedures significantly improves survival [175]. To the best of our knowledge, the intratumoral application has not yet been analyzed in clinical trials in GBM. The barrier to overcome in immunotherapies, and thus also in DCV, is TME. This could be a cause of immunosuppression, which correlates with the tumor size and surgical cytoreduction [176,177].

#### 3.8.4. Immune Checkpoint Inhibitors

The results of current immunotherapies encourage the use of immune checkpoint inhibitors (ICIs) such as antigen-programmed cell death (PD-1) antibody, which was first approved for treating malignant melanoma. Pardoll et al. showed that high expression of programmed cell death ligand 1 (PD-L1) in glioblastoma tumors is associated with poor survival, which makes it a therapeutic target for GBM. Immune checkpoints regulate the immune system by balancing inhibitory and regulatory effects. As a result, they obtain self-tolerance [178]. The most popular ones are the PD-1/PD-L1 pathway or cytotoxic T-lymphocyte-associated protein 4 (CTLA-4) [179,180,181]. Their discovery undoubtedly transformed the field of cancer immunotherapy [182,183]. ICIs targeting CTLA-4, PD-1, or indoleamine 2,3-dioxygenase 1 (IDO) yielded encouraging preclinical results in glioma mouse models [184,185]. However, they were unsuccessful in demonstrating the benefits of survival for patients with GBM. A few cases showed brilliant results, such as the case of a 60-year-old patient with recurrent GBM who received nivolumab for two years without any progression, toxicity, or need for corticosteroid treatment [186]. The patients received either nivolumab (anti-PD-1 antibody) or bevacizumab (anti-VEGF). The combination of these two drugs did not improve the mOS compared to other treatment arms [187]. Further addition of nivolumab to the standard therapy during the phase III trial (NCT02667587) evaluated anti-PD-1 in addition to TMZ plus RT versus TMZ plus RT in newly diagnosed MGMT-methylated GBM patients. It was conducted on 716 patients with newly diagnosed GBM and failed to meet its primary endpoint of improving mOS [188]. Even after these unsuccessful trials, there are new treatments that can yield better outcomes, such as a randomized, open-label pilot study from Cloughesy and colleagues [189]. In this study, an anti-PD-1 agent (pembrolizumab) was administered as a neoadjuvant drug to patients. Pembrolizumab before resection significantly improved mOS and PFS, with induction of TIL functional activation and production of an interferon (IFN-γ) response within the TME [189].

The second most popular ICI is CTLA-4; several phase I trials are evaluating its safety profile. The phase I trial NCT02794883 assessed the safety of anti-CTLA-4 and anti-PD-L1 antibodies in patients with recurring GBM. The phase I trial NCT02311920 evaluated the anti-CTLA-4 or anti-PD-1 drug in combination with TMZ for newly diagnosed patients with GBM or gliosarcoma. In summary, although ICIs have not demonstrated a significant benefit in the treatment of GBM in terms of improving overall survival, phase I trials assessing the safety of anti-CTLA-4 and anti-PD-L1 antibodies indicate ongoing research into their safety profile and potential efficacy. These preliminary analyses of safety and efficacy, despite limited success in improving clinical outcomes, highlight the need for further studies to better understand how these therapies can be optimally utilized in GBM treatment [190].

So far, ICIs as monotherapy are still ineffective in GBM, and there is no FDA-approved immunotherapy for GBM [187]. It is hoped that combination therapies will increase the efficiency of immunotherapies [191,192]. To increase the chances of finding the best therapy option, more and more inhibitors are being tested, such as CD-27, LAG-3, and IDO, as another immune checkpoint [193,194]. 

A new immune therapy strategy under study is the combination of anti-PD-1 adjuvant and neoadjuvant monotherapy with surgical resection. It was shown that supplying neoadjuvant and adjuvant anti-PD-1 improved mOS compared to patients that only received adjuvant anti-PD-1 in a group of 35 patients with recurrent, surgically resectable GBM [189]. Encouraging results were found in the phase II trial NCT02550249, in which patients received neoadjuvant anti-PD-1 and the adjuvant anti-PD-1 after surgical resection of GBM; this involved 30 patients, 27 with recurrent GBM, and 3 with newly diagnosed GBM. They showed that neoadjuvant anti-PD-1 enhanced the expression of chemokine transcription, increased the clonal diversity of TCR among the TILs, and increased the overall infiltration of tumor immune cells. However, no clinical benefits were found during this study.

Despite the effectiveness of immunotherapies, there are also have some strict limitations. Among their main obstacles are the BBB and intratumoral heterogeneity, which play a crucial role in immunotherapy resistance [195,196]. Others include the tumor microenvironment, which might suppress immune responses with T cells, myeloid-derived suppressor cells (MDSCs), and cytokines. To enhance immune activation, combination therapies might help. Additionally, immunotherapies might activate immune responses against healthy tissues, leading to the autoimmune reactions and other adverse effects.

#### 3.8.5. Oncolytic Viruses

Immunotherapy techniques often encounter difficulties connected with a highly suppressive TME. Therapies with oncolytic viruses (OVs) might be an interesting solution to this problem [197]. OVs use genetically engineered viruses, which can infect GBM cells selectively and replicate inside them, inducing the cells’ lysis and the release of tumor antigens [198]. Consequently, an adaptive antitumor response may be triggered thanks to the stimulation of antigen presenting cells (APCs). Many genres of OVs are successfully used in preclinical trials, including adenoviruses, herpes simplex viruses, parvoviruses, and measles viruses [199]. However, various clinical trials have also already been conducted, proving promising safety and efficacy of the OVs [198]. One of them is a phase I trial with Toca 511 by Cloughesy et al. It presented complete durable responses in about 20% of GBM patients receiving the virus intratumorally. Durable complete responses were found in some recurrent high-grade glioma patients treated with Toca 511 + Toca FC-PMC (nih.gov). Another virus, G47Δ, received conditional and time-limited approval for GBM treatment in Japan. It was a result of a phase II trial, which demonstrated an overall survival of 20.2 months after G47Δ initiation. Intratumoral oncolytic herpes virus G47∆ for residual or recurrent glioblastoma was tested in a phase 2 trial, PMC (nih.gov). A recent phase I clinical study tested the efficacy of CAN-3110, an oncolytic herpes virus, which was specifically modified to replicate preferentially in the tumor cells. A total of 41 glioblastoma patients were injected with the OVs. The treatment caused a significant increase in the number of lymphocytes in the tumor area. However, it was associated with longer survival only for herpes simplex virus 1 seropositive patients [197]. The outcomes seem promising, but deeper understanding of the therapy and its mechanism is still needed. 

## 4. Navigating the Blood–Brain Barrier and Immune Escape Mechanisms

### 4.1. Blood–Brain Barrier

The BBB is a semipermeable border between the circulatory system and CNS. The BBB comprises three main microvasculature elements: BBB-endothelial cells, astrocyte end-feet, and pericytes. They limit the paracellular flux of hydrophilic molecules across the BBB [200]. The BBB is permeable for gases but forms a barrier for larger molecules such as insulin and leptin [201]. Pathologically, numerous chemical mediators that increase BBB permeability have been released in the areas where the tumor is present [200].

The BBB is particularly disrupted in the cases of GBM due to the loss of expression of tight junctions’ protein claudin-1 in the microvessels, whereas claudin-5 and occludin are significantly downregulated in hyperplastic vessels [202]. In gliomas, SF/HGF appears to stimulate tumor cell motility, invasiveness, and protease expression. It also seems to be involved in the neovascularization process [203]. In high-grade gliomas, breakdown of the BBB produces the blood–brain tumor barrier (BBTB). This happens due to the high metabolic demands of high-grade glioma that create hypoxic areas. It triggers overexpression of VEGF and angiogenesis [204]. Thus, the BBTB and BBB create an obstacle for therapeutic agents [205]. However, some therapeutic agents may increase the penetration of the BBB, such as TMZ, mannitol, bradykinin agonists, and corticosteroids [206]. A potential novel solution for this could be nanocarrier-mediated therapy to overcome the problem of the BBB [207,208]. Recent advancements have demonstrated that nanoparticles can be conjugated with liposomes, dendrimers, metal ions, or polymeric micelles [207]. Some nanoparticles are tailored to engage in receptor-mediated transcytosis (attaching ligands that bind to the receptors on the endothelial cells of the BBB); however, others work through adsorption-mediated transcytosis using the surface charge of the BBB. This enhances the ability of drug-loaded compounds to effectively cross the BBB, providing new potential for overcoming GBM stem cell-mediated resistance to chemotherapy and radiation therapy. Combining chemotherapy with RNA interference (RNAi) molecules within the same nanocarrier can simultaneously inhibit tumor growth and reduce resistance mechanisms [209]. Nanocarriers might be used to deliver immune-modulating agents such as checkpoint inhibitors directly to the tumor site, which will help with modulating the tumor microenvironment, in turn boosting antitumor immunity [210].

### 4.2. Immune Escape Mechanisms

One of the key and most difficult obstacles to developing effective treatments for GBM is the tumor’s heterogeneity. This heterogeneity appears at different levels. Cellular heterogeneity is the difference between particular cells due to multiple transcriptional subtypes and subclones coexisting within the same tumor [56]. Spatial heterogeneity suggests that GBM exhibits varied enhancement and central necrosis, indicating a highly vascularized yet hypoxic core surrounded by a comparatively oxygenated periphery [211]. The other types of heterogeneity include differences between primary and recurrent GBM and various TMEs [212]. The abovementioned aspects imply the different resistance to cytotoxic chemotherapy, RT, and immunotherapies. The immunomodulating mechanism of the local microenvironment can affect the tumor itself; but, on the other hand, the tumor can modify the local environment and suppress the immune response [212]. Therefore, “cancer immunoediting” cells evolve to avoid immune-mediated elimination by leukocytes with antitumor properties [213]. Additional immunosuppressive properties may include the expression of PD-L1 or the secretion of suppressive cytokines, which also contribute to reduced immunogenicity. Membranous PD-L1 expression correlates with lymphocyte-rich tumor regions [214]. Interestingly, it may be used to define cancer response to immunotherapy. Furthermore, GBM tumors express the Fas ligand (CD95), which binds to Fas (CD95/APO1) on T cells, leading to their apoptosis, in turn enabling GBM cells to evade lysis by Fas-expressing T cells [215]. GBM tumors also secrete many other immunosuppressive molecules that enable immune evasion.

## 5. Conclusions and Future Perspectives

Despite many efforts and significant research, GBM still remains one of the most lethal cancers. Currently, various therapies improving GBM treatment aim at different cancer development points. Some of them are based on physical methods, such as proton beam therapy or tumor-treating fields. Additionally, there are also the achievements of today’s immunology and molecular biology—antibodies, inhibitor molecules, and modified immune cells. Additionally, more and more precise antigen selections and advances in biotechnology enable the creation of specific CAR constructs. CAR-T cells, which have huge potential to treat cancer, combine the exquisite antigen specificity, polyfunctionality, and potency of cellular immunity. Some CAR-NK cells could additionally become off-shelf products. Another emerging therapy is OV therapy, which utilizes viruses engineered to infect and destroy tumor cells. Some of these OVs are also designed to stimulate an immune response against the tumor.

In addition to these methods, gene editing techniques, particularly those involving CRISPR/Cas9, are being explored for their potential to correct genetic mutations associated with GBM or disrupt key pathways that contribute to tumor growth and therapy resistance. This genetic intervention could provide a pathway to more targeted and effective treatments. Further, the role of microRNAs in GBM pathogenesis is another area under intense study. Therapeutics targeting these microRNAs could potentially inhibit tumor growth or enhance the sensitivity of cancer cells to existing treatments, providing a novel approach to managing the disease.

Most of the methods described here increase median patient survival by only a few months. However, these are the first steps toward fully personalized treatment for GBM. One of the main challenges of today’s therapies is to create a targeted therapy, but also one that can penetrate the BBB. In describing such innovative treatments, it is also essential to consider their high cost and limited availability in many countries. Furthermore, many of these therapies do not offer significant benefits over standard treatment. However, their respective combinations are still unexplored, yet may be crucial in overcoming GBM.

## Data Availability

Not applicable.

## Supplementary Materials

The supporting information can be downloaded at: https://www.mdpi.com/article/10.3390/ijms25115774/s1.
