# Supplementary material for: Revolutionizing Glioblastoma Treatment: A Comprehensive Overview of Modern Therapeutic Approaches
Source: Int J Mol Sci. 2024 May 26;25(11):5774. doi: 10.3390/ijms25115774 (PMC11172387; doi:10.3390/ijms25115774)
Supplement: Supplementary file 1 [file ijms-25-05774-s001.zip › ijms-3020902-supplementary.pdf]

Table S1. Phase 2/3, 3 and 4 clinical trials of modern therapies for GBM. Glioblastoma (GBM), Gray (Gy), the relative biologic effectiveness (RBE), radiation therapy (RT), temozolomide (TMZ), standard of care (SOC), dendritic cells (DCs), O(6)-Methylguanine-DNA-methyltransferase (MGMT), ventriculoperitoneal shunt (VPs), intensity modulated radiation therapy (IMRT).

ClinicalTrials.gov Identifier	Title	Treatment	Phase	Number of patients	Dose
		**Physical methods**			
NCT04752280	Glioblastoma Radiotherapy Using IMRT or Proton Beams (GRIPS)	Radiation: Proton irradiation<br>Radiation: Photon irradiation	3	326	Proton irradiation<br>30 x 2 Gy(RBE) 33 x 1,8 Gy (RBE), or 15 x 2,67 Gy (RBE)
NCT05342883	GammaTile and Stupp in Newly Diagnosed GBM (GESTALT)	Radiation: GammaTile and TMZ and TTFs	4	61	No data available
NCT04536649	Proton and Heavy Ion Beam Radiation vs. Photon Beam Radiation for Newly Diagnosed Glioblastoma.	Radiation: Radiotherapy	3	369	Experimental carbon-ion radiation boost (15GyE/3F for residual lesion) priot to standard-dose proton radiation (60GyE/30F for high-risk area) or photon radiation (60Gy/30F for high-risk area) with concurrent temozolomide (75mg/m2, qd), then adjuvant temozolomide (150-200mg/m2, qd, D1-5, 28d/cycle).
NCT00916409	Effect of NovoTTF-100A Together with Temozolomide in Newly Diagnosed Glioblastoma M ultiforme (GBM)	NovoTTF-100A and TMZ	3	700	TMZ 150 mg/m2 or 200 mg/m2 daily
NCT01756729	Post-approval Study of NovoTTF-100A in Recurrent GBM Patients	NovoTTF-100A	4	13	Continuously
NCT00379470	Effect of NovoTTF-100A in Recurrent Glioblastoma Mu ltiforme (GBM)	NovoTFF-100A	3	236	No data available
NCT00689221	Cilengitide, Temozolomide, and Radiation Therapy in Treating Patients with Newly Diagnosed Glioblastoma and Methylated Gene Promoter Status (CENTRIC)	Proton beam therapy	3	545	
		**Pharmacological treatment**			
NCT00753246	Nimotuzumab in Adults with Glioblastoma Multifor ma	Nimotuzumab and TMZ and RT	3	150	No data available
NCT03025893	A Phase II/III Study of High-dose, Intermittent Sunitinib in Patients with Recurrent Glioblastoma Multiforme (STELLAR)	Sunitinib and Lomustine	2,3	100	Sunitinib 300 mg/week<br>Lomustine 110 mg/m2/6weeks

NCT00154375	Study of Imatinib Mesylate in Combination with Hydroxyurea Versus Hydroxyurea Alone as an Oral Therapy in Patients With Temozolomide Resistant Progressive Glioblastoma	Imatinib and Hydroxyurea	3	240	Imatinib 600 mg/day<br>Hydroxyurea 1000/1500mg/day
NCT02511405	A Phase 3, Pivotal Trial of VB-111 Plus Bevacizumab vs. Bevacizumab in Patients with<br>Recurrent Glioblastoma (GL OBE) (GLOBE)	VB-111 and bevacizumab	3	252	VB-111 1x10e13 VPs/2 months<br>10 mg/kg/2 weeks
NCT03776071	A Trial of Enzastaurin Plus Temozolomide During and Following Radiation Therapy in Patients with Newly Diagnosed Glioblastoma with or Without the Novel Genomic Biomarker, DGM1	Enzastaurin Hydrochloride and TMZ and RT	3	260	No data avilable
NCT00295815	Enzastaurin Versus Lomustine in Glioblastoma	Enzastaurin and Lomustine	3	397	1125 mg loading dose then 500 mg, oral, daily, 6 week cycles<br>Lomustine 100-130 mg/m2, oral once, every 6 weeks
NCT02573324	A Study of ABT-414 in Participants with Newly Diagnosed Glioblastoma (GBM) With Epidermal Growth Factor Receptor (EGFR) Amplification (Intellance1)	Depatuxizumab mafodotin and TMZ and RT	3	691	No data available
NCT02152982	Temozolomide With or Without Veliparib in Treating Patients with Newly Diagnosed Glioblastoma Multiforme	Veliparib and TMZ	2,3	447	No data available
NCT05326464	Tofacitinib in Recurrent GBM Patients	Tofacitinib	3	20	No data available
NCT06105619	A Study of PLB1001 Enteric Capsules in the Treatment of sGBM/IDH Mutant Glioblastoma Patients with the ZM Fusion Gene (FUGEN).	PLB1001 Enteric Capsules and TMZ and cisplatin and etoposide	3	84	PLB1001 300mg Bid, 28days/cycle<br>TMZ 100-150mg/m2/d, 7 days 1 to7 and days 15 to 22 of each 28-day cycle<br>cisplatin 80-100mg/m2/3 days, 28days/cycle etoposide 100mg/m2/d, 3days, 28 days/cycle
NCT03345095	A Phase III Trial of With Marizomib in Patients with Newly Diagnosed Glioblastoma (MIRAGE)	Marizomib and TMZ and RT	3	749	No data available

NCT02678975	Disulfiram in Recurrent Glioblastoma	Disulfiram	2, 3	88	Disulfiram 400mg daily, nutritional supplement with copper 2mg daily
NCT01290939	Bevacizumab and Lomustine for Recurrent GBM	Lomustine and bevacizumab	3	592	Lomustine 90 mg/m² every 6 weeks (cap. 160 mg) + bevacizumab 10 mg/kg every 2 weeks (at further progression treatment will be according to investigators discretion). In the absence of hematological toxicity > grade 1 during the first cycle the dose of lomustine can be escalated to 110 mg/m² (cap 200 mg) in their second cycle.
		**Immunotherapy**			
NCT04277221	ADCTA for Adjuvant Immunotherapy in Standard Treatment of Recurrent Glioblastoma Multiforme (GBM)	Autologous Dendritic Cell/ Tumor Antigen, ADCTA	3	118	Ten doses, including 2~4×10^7 cells for the 1st dose (double doses), and 1~2×10^7cells for the 2nd to 10th doses
NCT03548571	Dendritic Cell Immunotherapy Against Cancer Stem Cells in Glioblastoma Patients Receiving Standard Therapy	Dendritic cell immunization Drug: Adjuvant temozolomide and RT	2,3	60	RT 2 Gy x30
NCT01759810	Proteome-based Personalized Immunotherapy of Glioblastoma	Dendritic vaccine, allogeneic hematopoietic stem cells, cytotoxic lymphocytes Biological: Dendritic vaccine, autologous hematopoietic stem cells, cytotoxic lymphocytes	2,3	60	3 ml suspension of proteome-modified autologous hematopoietic cells in 0.9%NaCl solution/2 weeks 2 ml of individual dendritic vaccine in 4 points (shoulders and abdomen) 3 times every 14 days Meloxicam, 7.5mcg once a day
NCT02546102	Phase 3 Randomized, Double-blind, Controlled Study of ICT-107 in Glioblastoma	ICT-107	3	234	No data available
NCT04396860	Testing the Use of the Immunotherapy Drugs Ipilimumab and Nivolumab Plus Radiation Therapy Compared to the Usual Treatment (Temozolomide and Radiation Therapy) for Newly Diagnosed MGMT Unmethylated Glioblastoma	Ipilimumab and Nivolumab and TMZ and RT and Optune	2,3	485	No data available
NCT02667587	An Investigational Immunotherapy Study of Temozolomide Plus Radiation Therapy With Nivolumab or Placebo, for	Nivolumab and TMZ and RT	3	716	TMZ 75 or 150 or 200 mg/m2/day RT 2 Gy

	Newly Diagnosed Patients With Glioblastoma (GBM, a Malignant Brain Cancer) (CheckMate548)				
NCT04396860	Testing the Use of the Immunotherapy Drugs Ipilimumab and Nivolumab Plus Radiation Therapy Compared to the Usual Treatment (Temozolomide and Radiation Therapy) for Newly Diagnosed MGMT Unmethylated Glioblastoma	Nivolumab and TMZ and Ipilimumab and RT and TTF	2,3	159	RT 30 fractions over 6 weeks ipilimumab intravenously over 90 minutes once every four weeks for 4 doses nivolumab intravenously over 30 minutes every 2 weeks until disease progression
NCT02017717	A Study of the Effectiveness and Safety of Nivolumab Compared to Bevacizumab and of Nivolumab with or Without Ipilimumab in Glioblastoma Patients (CheckMate 143)	Nivolumab and TMZ and Ipilimumab	3	529	No data available
NCT02667587	An Investigational Immuno-therapy Study of Temozolomide Plus Radiation Therapy With Nivolumab or Placebo, for Newly Diagnosed Patients With Glioblastoma (GBM, a Malignant Brain Cancer) (CheckMate548)	TMZ and RT and Nivolumab	3	716	TMZ 75 mg/m^2 daily during RT, 4-week treatment break, 150 mg/m^2 Day 1-5 for Cycle 1 and increased to 200 mg/m^2 Day 1-5 for Cycle2-Cycle 6 as tolerated RT 2 gray units 5x/week x 6 weeks
NCT02617589	An Investigational Immuno-therapy Study of Nivolumab Compared to Temozolomide, Each Given With Radiation Therapy, for Newly-diagnosed Patients With Glioblastoma (GBM, a Malignant Brain Cancer) (CheckMate 498)	TMZ and RT and Nivolumab	3	560	No data available
NCT00045968	Study of a Drug [DCVax®-L] to Treat Newly Diagnosed GBM Brain Cancer (GBM)	DCVax®-L and Temodar and RT	3	348	On days 0, 10, and 20, then in months 2, 4, and 8 and months 12, 18, 24, and 30, with monthly TMZ as SOC. Each DCVax-L dose comprised 2.5 million DCs
NCT03149003	A Study of DSP-7888 Dosing Emulsion in Combination With Bevacizumab in Patients With Recurrent or Progressive Glioblastoma Following Initial Therapy	DSP-7888 Dosing Emulsion and Bevacizumab	3	221	DSP-7888 Dosing Emulsion every 7 ± 1 day for Doses 1 to 5, every 14 ± 3 days for Doses 6 to 15, and every 28 ± 7 days for Doses 16 and above Bevacizumab will be administered intravenously every 14 ± 3 days at 10 mg/kg
NCT01480479	Phase III Study of Rindopepimut/GM-CSF in Patients With Newly	Rindopepimut/GM-CSF and TMZ	3	745	CDX-110 500 mcg monthly GM-CSF 150 mcg monthly TMZ 150 to 200 mg/m2

	Diagnosed Glioblastoma (ACT IV)				
Others					
NCT03632135	Standard Chemotherapy vs. Chemotherapy Guided by Cancer Stem Cell Test in Recurrent Glioblastoma (CSCRGBM)	Chemotherapy and Cancer Stem Cell Test	3	78	Not apllicable
NCT02414165	The Toca 5 Trial: Toca 511 & Toca FC Versus Standard of Care in Patients With Recurrent High Grade Glioma (Toca5)	Toca 511 and Toca FC and TMZ and Lomustine and Bevacizumab	2,3	403	Toca 511 4 mL totally by injections and Toca FC 220 mg/kg/day orally for 7-day courses<br>Bevacizumab 10 mg/kg<br>Lomustine ingle oral dose of 110 mg/m2<br>TMZ 50/150/200 mg/m^2
NCT03291977	Interest of Fluorescein in Fluorescence-guided Resection of Gliomas (FLEGME) (FLEGME)	White light surgery with Fluoresceine Sodique Faure	3	51	Fluoresceine Sodique Faure given intravenously during the induction of the anesthesia, at the dose of 3mg/kg, diluted in 50mL of physiological serum, in 10 minutes.
